# Family Planning Uptake in Kagera and Mara Regions in Tanzania: A Cross-Sectional Community Survey

**DOI:** 10.3390/ijerph18041651

**Published:** 2021-02-09

**Authors:** Joseph Massenga, Rita Noronha, Bayoum Awadhi, Dunstan R. Bishanga, Oliva Safari, Lusekelo Njonge, Young-Mi Kim, Jos van Roosmalen, Thomas van den Akker

**Affiliations:** 1 Jhpiego Tanzania, Dar es Salaam 9170, Tanzania; rita.noronha@jhpiego.org (R.N.); bayoum@gmail.com (B.A.); lusekelo.njonge@jhpiego.org (L.N.); 2 Athena Institute, Vrije Universiteit, 1081HV Amsterdam, Noord-Holland, The Netherlands; J.J.M.van_Roosmalen@lumc.nl (J.v.R.); T.H.van_den_Akker@lumc.nl (T.v.d.A.); 3 School of Public Health and Social Sciences, Muhimbili University of Health and Allied Sciences, Dar es Salaam 11103, Tanzania; dbishanga@gmail.com; 4 Medical Teams International Kibondo, Dar es Salaam 47401, Tanzania; olivasafari@gmail.com; 5 Jhpiego, Baltimore, MD 21231, USA; young-mi.kim@jhpiego.org; 6 Department of Obstetrics and Gynaecology, Leiden University Medical Centre, 9300RC Leiden, Zuid-Holland, The Netherlands

**Keywords:** male partner, family planning, antenatal care, childbirth, community health worker, facility health care worker

## Abstract

In Tanzania, 27.1% of all women of reproductive age are currently using modern contraception and 16.8% have an unmet need for family planning. We therefore examined factors associated with family planning uptake after giving birth in two regions of Tanzania. The survey, which collected information beyond that collected in the Tanzania Demographic Health Survey, used a two-stage, stratified-cluster sampling design, conducted in April 2016 in Mara and Kagera regions in Tanzania. A total of 1184 women aged 15–49 years, who had given birth less than two years prior to the survey were included. Logistic regression mixed effect modelling was used to examine factors associated with family planning uptake. A total of 393 (33.2%) women used family planning methods and 929 (79%) required prior approval from their partners. Participation of men in utilization of maternal health care was low, where 680 (57.8%) women responded that their partners accompanied them to at least one antenatal care (ANC) counselling visit and 120 (10%) responded that their partners participated in family planning counselling. Women who did not want to disclose whether they had discussed family planning with their partners, strikingly had the highest percentage of using family planning methods after birth. Factors independently associated with family planning uptake included: having discussed family planning with the partner (aOR 3.22; 95% CI 1.99–5.21), having been counselled on family planning during antenatal care (aOR 2.68; 95% CI 1.78–4.05), having discussed family planning with a community health worker (CHW) (aOR 4.59; 95% CI 2.53–8.33) and with a facility health care worker (aOR 1.93; 95% CI 1.29–2.90), having primary or higher educational level (aOR 1.66; 95% CI 1.01–2.273), and being in union (aOR 1.86; 95% CI 1.02–3.42). Educational interaction with community and facility health workers, as well as having a supportive partner as facilitator increased uptake of family planning. This needs to be prioritized in regions with similar socio-cultural norms in Tanzania and beyond.

## 1. Introduction

Every year around 300,000 women and girls die worldwide in childbirth or from pregnancy-related complications, including abortion [1]. The majority of those occur in low- and middle-income countries (LMIC), especially among the poor and socially disadvantaged. A considerable proportion of deaths occurs in women with unintended pregnancies [2]. Access to family planning could avert more than 30% of maternal deaths and 10% of infant deaths, and women in LMIC are generally advised to space pregnancies more than two years apart [3]. Family planning after giving birth is considered a high-impact strategy to address unmet needs [4]. Demographic and Health Survey data from 27 countries showed that 95% of women who are within one year after birth want to avoid pregnancy for at least 24 months. Only 30%, however, use contraception during that time [5].

The maternal mortality ratio (MMR) in Tanzania has remained persistently high for over a decade with 556 deaths per 100,000 live births [6]. With multiple interventions and investments, family planning uptake has started to show an upward trend. Notwithstanding this trend, only 27.1% of women of reproductive age use modern contraceptives [6]. Unmet need of family planning has remained stable between 16.8% and 18.3% since 1999 [6,7].

Population growth rate in Tanzania is very high (2.7%), which has an effect on socio-economic development and the government needs to allocate more resources to counteract such population increase. The poverty rate stands at 28.2%, about one in four females aged ≥6 (24%) and one in five males aged ≥6 (19%) have no formal education, the unemployment rate stands at 9.7% and water availability at 45.2% of the households. Only 77% of urban and 55% rural households have three meals a day and annual expenditure on health is US$20.8 [8].

Family planning is one of the most cost-effective development interventions, with each dollar spent on family planning initiatives on average resulting in savings of US$6 on health, housing, water, and other public services. Therefore, family planning directly and indirectly contributes to the Sustainable Development Goals (SDGs) [9]. Hence, validating the rights of Tanzanian citizens to SDGs and recognizing the links between health and development, increasing uptake to quality, rights-based family planning care is a critical component of Tanzania’s journey to increase wealth and wellbeing for its citizens [10].

Antenatal care (ANC) provides a gateway to access reproductive, maternal, newborn, and child health care and a window of opportunity to promote the use of family planning after giving birth [11,12]. Family planning counselling within the realm of ANC could potentially increase uptake [13,14,15,16]. It may be beneficial to engage partners of women into such issues as contraception in case they have supportive attitudes towards family planning, since decision-making is often male-dominated. At the same time, uptake of family planning can improve women’s socioeconomic status and increase their level of agency [17,18,19,20,21,22,23,24,25]. In rural Tanzania, lack of communication among couples about family planning was previously identified as a barrier to uptake [26,27].

Studies have investigated other factors potentially influencing FP. Women may have a desire to live according to religious traditions as they found FP incompatible with their faith. This affirmed their responsibility to give birth to as many children as God would give them [28,29].There also are misinterpretation of Islamic teaching with regard to contraception, which is sometimes discouraged, and polygamy, which is sometimes still practiced and had a negative impact on FP use [30]. Sociocultural norms and values attached to marriage such as polygamy and extending family lineage remain impediments to using FP methods [31,32]. Increased access and use of FP have been seen among women with higher level of education and economic status [33,34,35,36]. Lack of knowledge on FP has been identified as barrier to use family planning [37,38,39]

Evidence is available from other countries on gender-related factors including discussion among couples about family planning, participation of male partners in reproductive health issues, and the effect of family planning counselling during ANC on uptake of contraceptives [12,13,17,22,23,40,41,42,43]. There is, however, paucity of evidence from Tanzania in this regard. The Tanzania Demographic Health Survey (TDHS-2015/2016) only explored gender-related power dynamics to a limited extent and additional studies are needed to generate local evidence to inform policy change, strengthen male involvement and enhance women’s autonomy in order to close the gaps in maternal health indicators including family planning uptake. Such efforts will contribute towards achieving the SDGs.

This study therefore aimed to assess whether family planning counselling by health workers during ANC and having discussed family planning with their partners are associated with the use of family planning among women within two years after childbirth. In addition to what is reported in the TDHS-2015/2016, study findings are expected to provide additional insight into potentially context-specific interventions to further promote the use of family planning after giving birth across the country.

## 2. Materials and Methods

### 2.1. Study Design and Setting

A cross-sectional study design was used to collect data in a survey conducted by the Maternal and Child Survival Program (MCSP). MCSP was a global project implemented in Kagera and Mara regions of Tanzania focusing on maternal and newborn health, family planning, malaria in pregnancy, and immunization. More details are reported in previous papers [33,34]. This household survey was conducted in April 2016 in Mara and Kagera regions in Tanzania.

Kagera region is located in the northwestern part of Tanzania, sharing borders with Uganda, Rwanda and Burundi. Kagera has a total population of 3,022,037, where women of reproductive age are 599,593 with a total fertility rate of 5.7 and a population growth rate of 3.2%. Mara region is located in the northeastern part of Tanzania, sharing borders with Kenya. Mara has a total population of 2,209,143, where women of reproductive age comprise 534,679 with a total fertility rate of 4.5 and a population growth rate of 2.5% [6,8]

### 2.2. Sampling and Population

The survey used a two-stage, stratified-cluster sampling design. In Kagera and Mara regions, divided into administrative districts, the 333 wards are broken down into enumeration areas (EAs). Each EA has approximately 100 households. In each region, 32 EAs were selected through the probability-proportional-to-size method. The first household was selected at random by dropping a pen into the generated EA household lists. Additional households were systematically selected from a list of households until we had interviewed at least 20 women who had recently given birth in one EA. If more than one eligible woman in a household consented to participate, all were interviewed. Since the survey applied a cluster sampling strategy, sample size was inflated with a design-effect of 1.5, adjusting for higher intra-cluster correlation, as well as 10% for effects of non-responses. A total of 1263 women of reproductive age were interviewed. Further details on the methods can be found in previous studies that used the same dataset [44,45,46].

Survey respondents were women aged 15–49 years who had given birth during the two years preceding the survey. We excluded 79 pregnant women who would not use any contraceptive method. A total of 1184 interviews with women were used in this analysis. Estimated sample size was assumed to detect an odds ratio (OR) of 2.2 with 80% power at 0.05 significance for the association, regarding risk factors between users of contraceptive methods versus non-users.

### 2.3. Measurements and Variables

Main outcome variable was use of modern family planning methods. These were defined as female and male sterilization, injectables, implants, pills, male and female condoms, and the lactational amenorrhoea method. Independent variables were age, level of education, living in union, parity, number of ANC visits, obtaining family planning counselling during ANC visits, having discussed family planning with community health workers (CHWs) or care providers in health facilities, having a partner accompanying her for ANC visits, having a partner participating in family planning counselling, being able to mention at least one family planning method, media exposure, partner’s approval of family planning use, having discussed family planning issues with partner, and women’s participation in decision making on health issues.

Family planning counselling was assessed on whether women received family planning counselling during ANC, where “0” was coded for those who did not receive counselling and “1” for those who did. Partners accompanying to ANC was defined as whether women were accompanied at least one ANC counselling visit. Exposure to media, without specifying the type of messaging, was assessed based on women’s reported exposure to at least one of the media (TV, Radio, and Newspaper). Being able to mention at least one family planning method spontaneously was assessed as women being able to mention any method, where 0 was coded for those who could not mention any method and 1 for those who could. Women’s participation in decision-making was a composite variable derived from six items assessing women’s participation in decision-making related to their own health and that of their children. These six items were (i) attending the doctor, (ii) accessing child immunizations, (iii) child-feeding practices, (iv) health care for sick children, (v) where to give birth, and (vi) where to seek care in case of pregnancy complications. Each of these items had six responses where in the first step of scale creation responses were grouped into three levels of ‘‘Alone’’, if women made decisions alone; ‘‘Jointly’’, if women made decisions jointly with their male partners or with other members of the family; and “Male partner alone or other members in the family”, if women did not participate in decision-making. In the second step the six items with the similar three levels as explained above were then grouped together. [46]. ANC attendance was assessed by whether women attended ANC and the number of visits (no visit, 1–3 or ≥4 visits). “Discussed family planning with community-based health workers” was defined as women who responded to having ever discussed family planning with a community health worker. “Discussed family planning with facility based health workers” was defined as women who responded to ever having discussed family planning issues with a health facility worker.

### 2.4. Data Collection

The original survey questionnaire was developed by the Child Survival Health Grants Program and MCSP [47]. Technical experts in the field of Reproductive Maternal, Newborn and Child health adapted it to reflect the Tanzanian context. The questionnaire was translated into Swahili. After local experts reviewed the Swahili tool, it was pilot-tested by research assistants and refined for better understanding. The survey was uploaded to the CommCare HQ mobile data collection platform on tablets.

To collect data, 30 female and male research assistants were recruited who were trained on research ethics, informed consent, sampling, recruitment, study, and other data collection procedures. Research assistants conducted face-to-face interviews in Swahili for a maximum of two hours with a participant and recorded women’s answers on tablets. Inbuilt skip patterns helped assure data quality where data collectors were instructed to skip some question(s) which were not supposed to be answered depending on previous responses. A data manager reviewed the data on a daily basis, and alerted study supervisors about errors to be addressed immediately.

### 2.5. Statistical Analysis

Analysis was conducted using Stata version 14 (StataCorp LLC, College Station, TX, USA). Frequency proportion tables were used to present categorical distributions of characteristics of participants. Bivariate analysis was used to examine crude relationships between use of modern family planning methods and a number of predictors, while, at multivariable level, the effects of multiple predictors were examined in relation to the use of modern family planning methods. Since this were nested data from multistage sampling of two levels, households are nested within clusters (Enumeration areas) meaning that each household belongs to one and only one Enumeration area and therefore a two levels mixed effect logistic regression model was fitted to the data. Accordingly, fixed effects, effects of independent variables in the use of modern family planning methods, were presented using odds ratios (ORs) with 95% confidence intervals (CIs), while random effects were presented using Intra-Class Correlations (ICC). The selection of variables added to the model was based on relevant variables previously reported in the literature to be associated with family planning and those of greater theoretical importance in our study setting. Variables were entered into multiple logistic regression model if crude analysis showed *p*-values < 0.20 [48].

### 2.6. Ethics Approval and Consent to Participate

All study participants provided oral consent before they participated. This study was reviewed and approved by the National Research and Ethics Committee (NatREC) with IRB Number NIMR/HQ/R.8a/vol.IX/2131, and the Johns Hopkins Bloomberg School of Public Health Institutional Review Board (IRB Number 5931).

## 3. Results

### 3.1. Characteristics of Participants

This analysis included 1184 women who had given birth two years prior to the survey and 169 (14.3%) women were within six months after childbirth. The majority was aged 25–34 years (512; 43.2%), had primary education and above (950; 80.2%) and lived in union with a partner (1004; 84.8%). Almost half had given birth to 2–4 children (544; 45.9%) (Table 1).

The proportion of women with any ANC visit was 94%, where more than half had attended four or more ANC visits (631; 53.3%) and less than two-thirds received family planning counselling during ANC (738; 62.3%). About a third (433; 36.6%) reported discussing family planning with care providers in health facilities whereas less than one in ten (102; 8.6%) did this with CHWs. Participation of men in utilization of maternal health services was low: less than third (680; 57.4%) responded that their partners accompanied them to at least one ANC counselling visit and 120(10.1%) women responded that their partners participated in family planning counselling (Table 1).

The majority of women were able to mention at least one family planning method (964; 82%) and less than two-thirds had exposure to at least one media source (659; 55.7%) (Table 1). Participants reported to having discussed family planning with their male partners in 328 (27.7%) whereas about 303 (25.6%) women opted not to disclose whether they consulted their male partners. Prior approval from their husband/partner to use family planning was required for 929 (79%) and about half of the women made decisions on health care jointly with their male partners or other family members (581; 49.1%) (Table 1).

### 3.2. Prevalence of Family Planning Use

**Figure 1:**
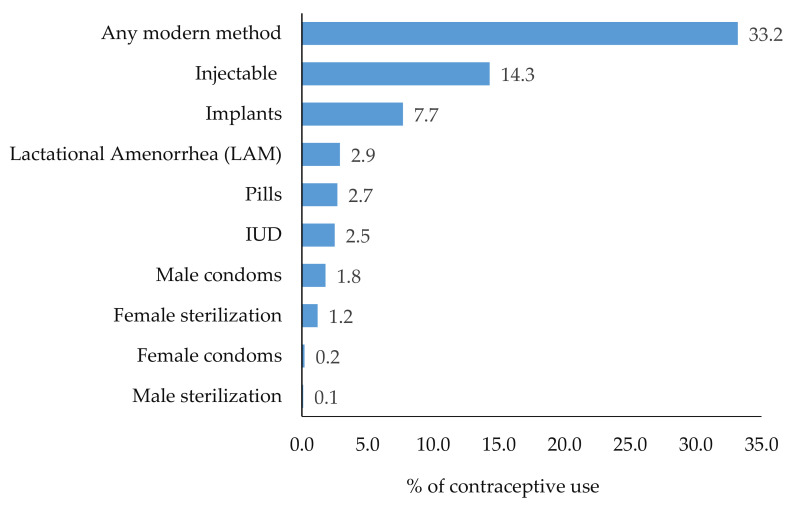
Proportion of women using contraceptive methods by contraceptive type in Kagera and Mara regions, Tanzania (*n* = 1184).

One third of the women were using family planning methods (393; 33.2%). Injectables (Depo-Provera) were the most frequently used method (14.3%), followed by implants (7.7%), where male sterilization (0.1%) and female condoms (0.2%) were almost never used (Figure 1).

### 3.3. Factors Associated with Family Planning Use

Women who had discussed family planning with CHWs, were four times more likely to use family planning compared to those who had not (aOR 4.59; 95% CI 2.53–8.33), but only few women (8.6%) got this counselling. Those who had discussed family planning with facility health workers (36.6%) were almost twice as likely to use family planning (aOR 1.93; 95% CI 1.29–2.90). Women who had received family planning counselling during ANC (62.3%) had more than twice the odds of using family planning methods compared to those who had not (aOR 2.68, 95%CI: 1.78–4.05). (Table 2). Moreover, women who had discussed family planning with their husband/partner, were about three times more likely to use family planning compared to women who had not (aOR 3.22, 95%CI: 1.99–5.21). Those who opted not to disclose whether or not they had discussed family planning with their husband/partner, had twenty-four times higher odds of family planning use compared to those who had not discussed this (aOR 24.19, 95%CI: 13.62–42.95) (Table 2).

## 4. Discussion

The purpose of this study was to assess whether family planning counselling by health workers during ANC and having discussed family planning with their partners are associated with increased use of family planning methods among women within two years after giving birth. About one-third who had recently given birth, reported using family planning after birth. Family planning counselling during ANC, having primary and above level of education, living in union, and family planning discussion with husband/male partners were associated with increased uptake of family planning. In this manner, our findings add importantly to those reported in the TDHS 2015/2016. Even though few women had a chance to discuss family planning with health care workers (both community and facility health workers), this was significantly associated with increased family planning use after giving birth.

Women who reported discussing family planning issues with their male partners were more likely to use family planning methods. Similar results were reported in India, Bangladesh, and Tanzania [23,37,38,39]. Involvement of male partners in reproductive health provides an opportunity for couples to discuss family issues including family planning [49]. When partners are open and supportive, and thus create an atmosphere for discussing family planning issues, women’s confidence and participation in decision-making may be promoted [42,43,44,45]. Lack of direct couple communication on family planning and spousal disapproval may decrease utilization of family planning [50,51]. Women who opted not to disclose whether they had discussed family planning with their husbands/partners had the highest odds of using family planning after childbirth. This can be explained by the fact that in Tanzania most women require husband/partner’s approval to use family planning, and due to fear of refusal or serious conflict, including violence or divorce if they went against their husbands’ wishes openly, most women use family planning without their partners’ knowledge [27]. Further analysis (see Appendix A revealed that these women are from families with lower wealth status and participate less in decision-making in health care for themselves and their children, undermining their autonomy and confidence to participate in health care [52,53,54]. Considering the variables reported in TDHS, our findings add important insight into the extent of gender inequity in relation to access to family planning, exploring power dynamics at play between women and their male partners. Having husbands/partners accompanying women to ANC, however, was not associated with increased family planning uptake. It is not merely the act of male partners accompanying women to the clinic that leads to adopting positive health behaviors, but rather the “content” of care couples receive [11]. It is likely that during such visits there was little or no discussion about family planning from health workers, hence, not encouraging dialogue between the couple [55]. If men are brought into a broad range of reproductive health care activities in such a way that they are supported as equitable partners and responsible parents, as well as clients in their own right, better outcomes are expected in reproductive health indicators such as contraception acceptance and continuation, safer sexual behavior, use of reproductive health care, and reduction of reproductive morbidity and mortality [56,57,58].

Women who reported receiving family planning counselling during ANC were more likely to use family planning after birth and this is in agreement with studies from India, Turkey, and Mexico [59]. For effective family planning use after birth, counselling should start during pregnancy in ANC and this will give women an opportunity to receive health information that extends beyond pregnancy [9,10,13,49,50]. Our findings add to the knowledge about the interaction between women and facility health workers with regard to family planning counselling during ANC and suggest that family planning counselling should be offered to all women during ANC visits to increase uptake after birth.

Women who discussed family planning with CHWs were four times more likely to use family planning and CHWs are able to build trusted relationship in the community. Being originally from the same community and making frequent household visits enhance familiarity that enables more time for health talks and women are free to discuss and express their concerns, This gives CHWs more opportunity and authority to provide relevant information about FP methods promoting family planning uptake [60]. Although only less than one in ten women discussed family planning with CHWs, their odds of FP uptake were higher. If there would have been an opportunity for all women to discuss FP with CHWs, uptake after birth would have been much higher. This implies that more efforts should also be invested in community based interventions to reach the grassroot level with appropriate information to increase FP uptake [61,62,63,64].

Discussing family planning with health workers increased the use of family planning after birth as has been shown in Kenya and Pakistan [55,56]. Worrying, however, is the finding that contrary to national guidelines half of the women did not discuss these issues with health workers [65]. Health care workers should be supportive and open up discussions in a safe and correct manner as this may help to clarify women’s misconceptions and concerns with family planning methods. It may enable women to choose the methods that best match their needs [66,67,68,69]. The finding of fewer women having had the opportunity to discuss FP with health workers, could be due to barriers faced by health care workers including work overload, lack of private space for counselling and lack of Information, Education and Communication (IEC) materials [70]. When these barriers are addressed, women will get an opportunity to receive adequate counselling and this may improve family planning uptake [70,71].

We also looked into regional differences as appears in the supplementary table in Appendix B. In this study, however, Kagera and Mara were taken as one population and the study was not powered to detect differences between the two regions. A next study is recommended to power it to detect regional differences and its reasons. We have witnessed many women starting with FP after having received counselling during pregnancy. We recommend a future qualitative study to find out what made women actually start FP after getting counselling during pregnancy and what could be the difference between counselling by facility or community health workers.

Addition of variables which could not be found in TDHS 2015/2016 provided increased insight into gender inequity, power dynamics at play between women and their partners, barriers faced by women, male involvement, and coverage of family planning counseling during ANC as key determinants of family planning uptake after birth. This comprises crucial grassroot information with important policy implications that could not have been identified based on TDHS data only.

### Study Strengths and Limitations

Strengths of this study are a stratified cluster sampling that accounted for between and within group variations on the use of family planning; and large sample sizes to represent the populations of Kagera and Mara regions. Although not powered enough, Appendix A still gives some insight in the differences between the two regions. Since this study used a cross-sectional design, no causal inferences can be drawn. It is possible that women who took up family planning methods were more likely to discuss family planning with their husbands/partners, simply because they had husbands/partners more likely to support them in these matters. In this way, it is not the fact that they had discussed the matter that led to family planning uptake, but rather the type of relationship of the couple and attitudes of the husband that enabled them to discuss as well as take up family planning. Moreover, the sample was drawn from only two out of twenty six regions of Tanzania, thus limiting generalizability to the whole country. The tool was not translated back to English from Swahili and cultural context validation not done.

## 5. Conclusions

Only one in three women who had recently given birth reported using family planning methods in Kagera and Mara regions in Tanzania. Such low use of family planning jeopardizes any effort to address maternal and child health challenges that remain persistent in these and other regions with a similar context in Tanzania. Addressing such challenges call for factors that influence use of family planning in this population. There is a relatively high number of women who did not want to disclose involvement of their partners’ role and those women had the highest odds of using family planning methods. Therefore, meaningful engagement of male partners to support their female partners in reproductive health can be of added value in these regions. Similarly, improved interaction of women with CHWs at community level and strengthening health talks at the facility levels have the potential to increase family planning uptake in these and other regions with similar socio-cultural norms in Tanzania. Such efforts should be streamlined within existing ANC opportunities. In addition to key information reported in the TDHS 2015–2016, these findings add to the knowledge about key determinants of family planning use after giving birth in Kagera and Mara regions in Tanzania.

## Figures and Tables

**Table 1:** Characteristics of participantsin Kagera and Mara regions, Tanzania (*n* = 1184).

Variable	*n*	%
Age *		
15–24	496	41.9
25–34	512	43.2
35+	176	14.9
Education *		
No education	234	19.8
Primary and above	950	80.2
State of being in union with male Partner *		
Not in union	143	12.1
In union	1004	84.8
Parity *		
1	246	20.8
2–4	544	45.9
5+	394	33.3
**Maternity Care Utilization**		
Antenatal care attendance *		
Never	68	5.7
1–3 visits	485	41.0
4+ visits	631	53.3
Family planning counselling during antenatal care **		
No	444	37.5
Yes	738	62.3
Discussed family planning with community health worker *		
No	1082	91.4
Yes	102	8.6
Discussed family planning with facility based health worker *		
No	751	63.4
Yes	433	36.6
Partner accompany to ANC **		
No	497	42.0
Yes	680	57.4
Partner participated in family planning counselling **		
No	1064	89.9
Yes	120	10.1
**Knowledge On Family Planning**		
Being able to mention at least one family planning method *		
Not able to mention any method	211	17.8
Mentioned at least one method	964	81.4
Exposure to media *		
No exposure to tv, radio & newspapers	291	24.6
Exposed to at least one media source	659	55.7
**Gender Related Factors**		
Discuss family planning with partner **		
No	553	46.7
Yes	328	27.7
Opted not to disclose whether they discussed or not	303	25.6
Need husband/partner’s approval to use family planning **		
No	248	21.0
Yes	929	78.5
Decision-making in healthcare *		
Woman alone	175	14.8
Jointly	581	49.1
Male partner alone/others alone	421	35.6

Not all variables add up to the total population because of missing observations. * Similar to variables reported in TDHS 2015/2016. ** Not reported in TDHS 2015/2016.

**Table 2:** Bivariate and multivariate logistic regression analysis examining factors associated with modern family planning use in Kagera and Mara regions, Tanzania (*n* = 1067).

Variable	Family Planning Use		Bivariate		Multivariate
	Yes	%	OR (95% CI)	*p*-Value	aOR (95% CI)
**Age**					
15–24	155	31.3	1		1
25–34	176	34.4	1.17(0.88–1.54)	0.29	1.34 (0.91–1.99)
35+	47	26.7	0.78 (0.51–1.17)	0.23	0.91 (0.53–1.58)
**Education**					
No education	56	23.9	1		1
Primary and above	322	33.9	1.52 (1.06–2.19)	0.02	1.66 (1.01–2.73)
State of being in union/or not *					
Not in union	37	25.9	1		1
In union	335	33.4	1.61 (1.04–2.47)	0.03	1.86 (1.02–3.42)
**Region**					
Mara	150	25.3	1		1
Kagera	228	38.6	1.96(1.34–2.89)	0.001	1.65 (0.89–3.04)
**Parity**					
1	72	29.3	1		
2–4	188	34.6	1.31 (0.92–1.87)	0.13	
5+	118	30.0	1.11 (0.76–1.62)	0.58	
**Antenatal care attendance**					
Never	24	35.3	1		
1–3	137	28.3	0.83 (0.47–1.47)	0.53	
4+	217	34.4	1.05 (0.60–1.83)	0.88	
Family planning counseling during antenatal care *					
No	84	18.9	1		1
Yes	293	39.7	3.01 (2.22–4.09)	<0.001	2.68 (1.78–4.05)
**Discussed family planning with community health worker**					
No	317	29.3	1		1
Yes	61	59.8	3.42 (2.18–5.38)	<0.001	4.59 (2.53–8.33)
**Discussed family planning with facility health worker**					
No	167	22.2	1		1
Yes	211	48.7	3.88 (2.91–5.18)	<0.001	1.93 (1.29–2.90)
Partner company to antenatal care *					
No	129	26	1		1
Yes	245	36	1.43 (1.08–1.89)	0.01	1.26 (0.84–1.90)
**Partner participated in family planning counseling**					
No	307	28.9	1		1
Yes	71	59.2	4.03 (2.62–6.19)	<0.001	0.97 (0.56–1.67)
**Being able to mention at least one family planning method**					
Not able to mention any family planning method	50	23.7	1		1
Mentioned at least one family planning method	328	34.0	1.63 (1.12–2.37)	0.01	1.10 (0.66–1.83)
Exposure to media *					
No exposure to tv, radio & newspapers	85	29.2	1		
Exposed to atleast one media source	237	36.0	1.19 (0.85–1.66)	0.32	
**Discussed family planning with partner**					
No	63	11.4	1		1
Yes	119	36.3	3.72 (2.52–5.49)	<0.001	3.22 (1.99–5.21)
Opted not to disclose	196	64.7	27.51 (17.53–43.17)	<0.001	24.19 (13.62–42.95)
Need husband/partner approval to * use family planning					
No	67	27	1		
Yes	307	33.1	1.25 (0.89–1.74)	0.20	
Decision-making in healthcare *					
Woman alone	126	33.7	1		
Jointly	86	23.0	0.97 (0.67–1.41)		0.87
Male partner alone/others alone	162	43.3	1.01 (0.73–1.40)		0.94
**Random effects**					
(ς_u_)^2^		0.94			
ICC		0.23			
**Model fitness**					
Likelihood value		−448.67			

* Do not add up to 378 for family planning use because of missing observations.

## Data Availability

The data presented in this study are available on request from the corresponding author. The data are not publicly available due local IRB regulation.

## Appendix A

**Table A1:** Further analysis of women discussed family planning with male partner in Kagera and Mara regions, Tanzania.

Variable	Discussed Family Planning with Male Partner			Chi-Square *p*-Value
	No	Yes	Opted Not to Disclose	
	*n* (%)	*n* (%)	*n* (%)	
**Education**				*p* = 0.124
No Education	54 (23.1)	122 (52.1)	58 (24.8)	
Primary and above	274 (28.8)	431 (45.4)	245 (25.8)	
**Age category**				*p* = 0.559
15–24	143 (28.8)	230 (46.4)	123 (24.4)	
25–34	138 (27.0)	233 (45.5)	141 (27.5)	
35+	47 (26.7)	90 (51.1)	39 (22.2)	
**Parity**				*p* = 0.386
1	65 (26.4)	126 (51.2)	55 (22.4)	
2 to 4	156 (28.7)	239 (43.9)	149 (27.4)	
5+	107 (27.2)	188 (47.7)	99 (25.1)	
**Wealth**				*p* < 0.001
Poor	114 (29.8)	163 (42.5)	106 (27.7)	
Middle	129 (33.2)	162 (41.8)	97 (25.0)	
Rich	75 (19.9)	206 (54.8)	95 (25.3)	
**Decision making on health**				*p* < 0.01
Alone	31 (17.7)	105 (60.0)	39 (22.3)	
Jointly	176 (30.3)	254 (43.7)	151 (26.0)	
Male partner/others alone	121 (28.7)	194 (46.1)	106 (25.2)	

## Appendix B

**Table A2:** Supplementary Table Showing Regional Differences Using Chi-Square Test in Kagera and Mara Regions, Tanzania.

Variable	Total		Kagera		Mara		Chi-Square *p*-Value
	n	%	n	%	n	%	
**Women reported male partner Participated on FP counseling**							0.33
No	1064	89.9	538	90.7	526	89.0	
Yes	120	10.1	55	9.3	65	11.0	
**Awareness on FP methods**							<0.001
No	211	18.0	137	23.3	74	12.6	
Yes	964	82.0	452	76.7	512	87.4	
**FP discussion with male partner**							<0.001
No	328	27.7	130	21.9	198	33.5	
Yes	550	46.5	326	55.0	224	37.9	
Don’t want	306	25.8	137	23.1	169	28.6	
**Discuss FP with Community Health Workers**							0.82
No	1082	91.4	543	91.6	539	91.2	
Yes	102	8.6	50	8.4	52	8.8	
**Discuss FP with health Facility worker**							0.82
No	751	63.4	422	71.2	329	55.7	
Yes	433	36.6	171	28.8	262	44.3	
**FP Counselling at ANC**							<0.001
No	444	37.6	258	43.6	186	31.5	
Yes	738	62.4	334	56.4	404	68.5	
**Parity**							0.00
1	246	20.8	101	17.0	145	24.5	
2 to 4	544	46.0	276	46.5	268	45.4	
5+	394	33.3	216	36.4	178	30.1	
**State of being in union**							0.75
In-union	1004	87.5	499	87.9	505	87.2	
Not in-union	143	12.5	69	12.2	74	12.8	
**Education Status**							0.012
No education	234	19.8	101	17.0	133	22.5	
Primary and above	950	80.2	492	83.0	458	77.5	
**Age category**							0.07
15–24	496	41.9	255	43.0	241	40.8	
25–34	512	43.2	239	40.3	273	46.2	
35+	176	14.9	99	16.7	77	13.0	
**Decision making on health care**							0.20
Women alone	175	14.9	94	15.9	81	13.8	
Jointly	581	49.4	276	46.8	305	56.9	
Male partner alone/others alone	421	37.8	220	37.3	201	34.2	

## References

[1] (2015). WHO/Jhpiego Postnatal Care for Mothers and Newborns Highlights from the World Health Organization 2013 Guidelines. Postnatal Care Guidel.

[2] Sedgh G., Singh S., Hussain R. (2014). Intended and unintended pregnancies worldwide in 2012 and recent trends. Stud. Fam. Plann..

[3] Cleland J., Bernstein S., Ezeh A., Faundes A., Glasier A., Innis J. (2006). Family planning: The unfinished agenda. Lancet.

[4] WHO (2013). Postnatal Care of the Mother and Newborn 2013.

[5] Ross J.A., Winfrey W.L. (2001). Contraceptive Use, Intention to Use and Unmet Need during the Extended Postpartum Period. Int. Fam. Plan. Perspect..

[6] Ministry of Health, Community Development, Gender, Elderly and Children (MoHCDGEC), Ministry of Health (MoH), National Bureau of Statistics (NBS), Office of Chief Government Statistician (OCGS), ICF 2016 Tanzania Demographic and Health Survey and Malaria Indicator Survey (TDHS-MIS) 2015-16. Dar es Salaam, Tanzania, and Rockville, MD, USA, 2016. www.nbs.go.tz.

[7] National Bureau of Statistics (NBS) and ICF Macro Tanzania Demographic and Health Survey 2010. Natl. Bur. Stat. Dar es Salaam, Tanzania ICF Macro Calverton, Maryland, USA 2011. www.nbs.go.tz.

[8] National Bureau of Statistics (NBS) (2019). Tanzania in Figures 2018. Dodoma. www.nbs.go.tz.

[9] Moreland S., Talbird S., U.S. Agency for International Development (USAID) (2006). Achieving the Millennium Development Goals: The Contribution of Fulfilling the Unmet Need for Family Planning.

[10] Ministry of Health Community Development Gender Elderly and Children (MoHCDGEC) Tanzania (2019). National Family Planning Costed Implementation Plan 2019–2023.

[11] WHO (2016). WHO Recommendations on Antenatal Care for a Positive Pregnancy Experience.

[12] Do M., Hotchkiss D. (2013). Relationships between antenatal and postnatal care and post-partum modern contraceptive use: Evidence from population surveys in Kenya and Zambia. BMC Health Serv. Res..

[13] Agha S., Williams E. (2016). Does the antenatal care visit represent a missed opportunity for increasing contraceptive use in Pakistan? An analysis of household survey data from Sindh province. Health Policy Plan..

[14] Keogh S.C., Urassa M., Kumogola Y., Kalongoji S., Kimaro D., Zaba B. (2015). Postpartum Contraception in Northern Tanzania: Patterns of Use, Relationship to Antenatal Intentions, and Impact of Antenatal Counseling. Stud. Fam. Plann..

[15] Vural F., Vural B., Cakıroglu Y. (2016). The effect of combined antenatal and postnatal counselling on postpartum modern contraceptive use: Prospective case-control study in Kocaeli, Turkey. J. Clin. Diagn. Res..

[16] Dona A., Abera M., Alemu T., Hawaria D. (2018). Timely initiation of postpartum contraceptive utilization and associated factors among women of child bearing age in Aroressa District, Southern Ethiopia: A community based cross-sectional study. BMC Public Health.

[17] Hartmann M., Gilles K., Shattuck D., Kerner B., Guest G. (2012). Changes in couples’ communication as a result of a male-involvement family planning intervention. J. Health Commun..

[18] Osuafor G.N., Maputle S.M., Ayiga N. (2018). Corrigendum: Factors related to married or cohabiting women’s decision to use modern contraceptive methods in Mafikeng, South Africa. Afr. J. Prim. Health Care Fam. Med..

[19] Babalola S., Oyenubi O., Speizer I.S., Cobb L., Akiode A., Odeku M. (2017). Factors affecting the achievement of fertility intentions in urban Nigeria: Analysis of longitudinal data. BMC Public Health.

[20] Kassa M., Abajobir A.A., Gedefaw M. (2014). Level of male involvement and associated factors in family planning services utilization among married men in Debremarkos town, Northwest Ethiopia. BMC Int. Health Hum. Rights.

[21] Khan R.N.J., Hashim S.M., Nawi A.M., Siraj H.H. (2018). Factors associated with ever used of modern contraception among married men attending a primary healthcare clinic. Med. J. Malays..

[22] Kamal N. (2000). The influence of husbands on contraceptive use by Bangladeshi women. Health Policy Plan..

[23] Abraha T.H., Belay H.S., Welay G.M. (2018). Intentions on contraception use and its associated factors among postpartum women in Aksum town, Tigray region, northern Ethiopia: A community-based cross- sectional study. Reprod. Health.

[24] Asaolu I.O., Okafor C.T., Ehiri J.C., Dreifuss H.M., Ehiri J.E. (2017). Association between Measures of Women’s Empowerment and Use of Modern Contraceptives: An Analysis of Nigeria’s Demographic and Health Surveys. Front. Public Health.

[25] Mboane R., Bhatta M.P. (2015). Influence of a husband’s healthcare decision making role on a woman’s intention to use contraceptives among Mozambican women. Reprod. Health.

[26] Mosha I., Ruben R., Kakoko D. (2013). Family planning decisions, perceptions and gender dynamics among couples in Mwanza, Tanzania: A qualitative study. BMC Public Health.

[27] Schuler S.R., Rottach E., Mukiri P. (2011). Gender norms and family planning decision-making in Tanzania: A qualitative study. J. Public Health Afr..

[28] Sundararajan R., Yoder L.M., Kihunrwa A., Aristide C., Kalluvya S.E., Downs D.J., Mwakisole A.H., Downs J.A. (2019). How gender and religion impact uptake of family planning: Results from a qualitative study in Northwestern Tanzania. BMC Women’s Health.

[29] Srikanthan A., Reid R.L. (2008). Religious and Cultural Influences on Contraception. J. Obstet. Gynaecol. Can..

[30] Abdi B., Okal J., Serour G., Temmerman M. (2020). “Children are a blessing from God”—A qualitative study exploring the socio-cultural factors influencing contraceptive use in two Muslim communities in Kenya. Reprod. Health.

[31] Kabagenyi A., Reid A., Ntozi J., Atuyambe L. (2016). Socio-cultural inhibitors to use of modern contraceptive techniques in rural Uganda: A qualitative study. Pan Afr. Med. J..

[32] De Vargas Nunes Coll C., Ewerling F., Hellwig F., De Barros A.J.D. (2019). Contraception in adolescence: The influence of parity and marital status on contraceptive use in 73 low-and middle-income countries. Reprod. Health.

[33] Apanga P.A., Kumbeni M.T., Ayamga E.A., Ulanja M.B., Akparibo R. (2020). Prevalence and factors associated with modern contraceptive use among women of reproductive age in 20 African countries: A large population-based study. BMJ Open.

[34] Mekonnen W., Worku A. (2011). Determinants of low family planning use and high unmet need in Butajira District, South Central Ethiopia. Reprod. Health.

[35] Kanma-Okafor O.J., Asuquo E.J.I.M., Balogun M.R.A.O. (2019). Utilisation and Preferences of Family Planning Services among Women in Ikosi-Isheri, Kosofe Local Government Area, Lagos, Nigeria. Niger. Postgrad Med. J..

[36] Agadjanian V., Hayford S.R., Luz L., Yao J. (2015). Bridging user and provider perspectives: Family planning access and utilization in rural Mozambique. Int. J. Gynecol. Obstet..

[37] Schultz C., Larrea N., Celada M., Heinrichs G. (2018). A Qualitative Assessment of Community Attitudes and Barriers to Family Planning Use in the Trifinio Region of Southwest Guatemala. Matern. Child Health J..

[38] Semachew Kasa A., Tarekegn M., Embiale N. (2018). Knowledge, attitude and practice towards family planning among reproductive age women in a resource limited settings of Northwest Ethiopia. BMC Res. Notes.

[39] Mustafa G., Azmat S.K., Hameed W., Ali S., Ishaque M., Hussain W., Ahmed A., Munroe E. (2015). Family Planning Knowledge, Attitudes, and Practices among Married Men and Women in Rural Areas of Pakistan: Findings from a Qualitative Need Assessment Study. Int. J. Reprod. Med..

[40] Kabagenyi A., Jennings L., Reid A., Nalwadda G., Ntozi J., Atuyambe L. (2014). Barriers to male involvement in contraceptive uptake and reproductive health services: A qualitative study of men and women’s perceptions in two rural districts in Uganda. Reprod. Health.

[41] Spagnoletti B.R.M., Bennett L.R., Kermode M., Wilopo S.A. (2018). “I wanted to enjoy our marriage first... but I got pregnant right away”: A qualitative study of family planning understandings and decisions of women in urban Yogyakarta, Indonesia. BMC Pregnancy Childbirth.

[42] Izugbara C., Ibisomi L., Ezeh A.C., Mandara M. (2010). Gendered interests and poor spousal contraceptive communication in Islamic northern Nigeria. J. Fam. Plan. Reprod. Health Care.

[43] Mansor M., San S.O., Abdullah K.L. (2015). Prevalence of Family Planning Practices among Women Influenced by Husband’s Socio Demography and Decision Making. J. Sains Kesihat. Malays..

[44] Bishanga D.R., Massenga J., Mwanamsangu A.H., Kim Y.M., Eorge J., Kapologwe N.A., Zoungrana J., Rwegasira M., Kols A., Hill K. (2019). Women’s experience of facility-based childbirth care and receipt of an early postnatal check for herself and her newborn in Northwestern Tanzania. Int. J. Environ. Res. Public Health.

[45] Jhpiego, ICF/Macro International Inc., John Snow Inc. (2015). Save the Children. Maternal and Child Survival Program (MCSP); Year 1 Implementation Plan June 2014–September 2015.

[46] Bishanga D.R., Drake M., Kim Y.M., Mwanamsangu A.H., Makuwani A.M., Zoungrana J., Lemwayi R., Rijken M.J., Stekelenburg J. (2018). Factors associated with institutional delivery: Findings from a cross-sectional study in Mara and Kagera regions in Tanzania. PLoS ONE.

[47] United States Agency for International Development (USAID) Bureau for Global Health (2002). Child Survival and Health Grants Program.

[48] Bursac Z., Gauss C.H., Williams D.K., Hosmer D.W. (2008). Purposeful selection of variables in logistic regression. Source Code Biol. Med..

[49] Shahidul Islam M., Shafiul Alam M., Mahedi Hasan M. (2014). Inter-spousal communication on family planning and its effect on contraceptive use and method choice in Bangladesh. Asian Soc. Sci..

[50] Do M., Kurimoto N. (2012). Women’s empowerment and choice of contraceptive methods in selected African countries. Int. Perspect. Sex. Reprod. Health.

[51] Wolff B., Blanc A.K., Ssekamatte-Ssebuliba J. (2000). The Role of Couple Negotiation in Unmet Need for Contraception and the Decision to Stop Childbearing in Uganda. Stud. Fam. Plann..

[52] Rizkianti A., Afifah T., Saptarini I., Rakhmadi M.F. (2020). Women’s decision-making autonomy in the household and the use of maternal health services: An Indonesian case study. Midwifery.

[53] Ghose B., Feng D., Tang S., Yaya S., He Z., Udenigwe O., Ghosh S., Feng Z. (2017). Women’s decision-making autonomy and utilisation of maternal healthcare services: Results from the Bangladesh Demographic and Health Survey. BMJ Open.

[54] Ameyaw E.K., Tanle A., Kissah-Korsah K., Amo-Adjei J. (2016). Women’s Health Decision-Making Autonomy and Skilled Birth Attendance in Ghana. Int. J. Reprod. Med..

[55] Kim Y.M., Kols A., Mwarogo P., Awasum D. (2000). Differences in counseling men and women: Family planning in Kenya. Patient Educ. Couns..

[56] Wegner M.N., Landry E., Wilkinson D., Tzanis J. (1998). Men as Partners in Reproductive Health: From Issues to Action. Int. Fam. Plan. Perspect..

[57] Bustamante-Forest R., Giarratano G. (2004). Changing men’s involvement in reproductive health and family planning. Nurs. Clin. N. Am..

[58] Kejela G. (2017). The Role of Male Involvement in Modern Family Planning Utilization and Associated Factors in Arba Minch Town, Gamo Gofa Zone, Ethiopia. Eur. J. Prev. Med..

[59] Barber S.L. (2007). Family Planning Advice and Postpartum Contraceptive Use Among Low-Income Women in Mexico. Int. Fam. Plan. Perspect..

[60] Frumence G., Goodman M., Chebet J.J., Mosha I., Bishanga D., Chitama D., Winch P.J., Killewo J., Baqui A.H. (2019). Factors affecting early identification of pregnant women by community health workers in Morogoro, Tanzania. BMC Public Health.

[61] Mazzei A., Ingabire R., Mukamuyango J., Nyombayire J., Sinabamenye R., Bayingana R., Parker R., Tichacek A., Easter S.R., Karita E. (2019). Community health worker promotions increase uptake of long-acting reversible contraception in Rwanda. Reprod. Health.

[62] Brooks M.I., Johns N.E., Quinn A.K., Boyce S.C., Fatouma I.A., Oumarou A.O., Sani A., Silverman J.G. (2019). Can community health workers increase modern contraceptive use among young married women? A cross-sectional study in rural Niger. Reprod. Health.

[63] Lutalo T., Kigozi G., Kimera E., Serwadda D., Wawer M.J., Zabin L.S., Gray R.H. (2010). A randomized community trial of enhanced family planning outreach in Rakai, Uganda. Stud. Fam. Plann..

[64] Tappis H., Kazi A., Hameed W., Dahar Z., Ali A., Agha S. (2015). The role of quality health services and discussion about birth spacing in postpartum contraceptive use in Sindh, Pakistan: A multilevel analysis. PLoS ONE.

[65] Ministry of Health and Social Welfare (MOHSW) (2013). National Family Planning Guidelines and Standards.

[66] Belda S.S., Haile M.T., Melku A.T., Tololu A.K. (2017). Modern contraceptive utilization and associated factors among married pastoralist women in Bale eco-region, Bale Zone, South East Ethiopia. BMC Health Serv. Res..

[67] Lakew Y., Reda A.A., Tamene H., Benedict S., Deribe K. (2013). Geographical variation and factors influencing modern contraceptive use among married women in Ethiopia: Evidence from a national population based survey. Reprod. Health.

[68] Medhanyie A., Spigt M., Kifle Y., Schaay N., Sanders D., Blanco R. (2012). The role of health extension workers in improving utilization of maternal health services in rural areas in Ethiopia: A cross sectional study. BMC Health Serv. Res..

[69] Kabagenyi A., Ndugga P., Wandera S.O., Kwagala B. (2014). Modern contraceptive use among sexually active men in Uganda: Does discussion with a health worker matter?. BMC Public Health.

[70] Puri M.C., Maharjan M., Pearson E., Pradhan E., Dhungel Y., Khadka A., Shah I.H. (2018). Delivering postpartum family planning services in Nepal: Are providers supportive?. BMC Health Serv. Res..

[71] LeFevre A., Mpembeni R., Kilewo C., Yang A., An S., Mohan D., Mosha I., Besana G., Lipingu C., Callaghan-Koru J. (2018). Program assessment of efforts to improve the quality of postpartum counselling in health centers in Morogoro region, Tanzania. BMC Pregnancy Childbirth.

